# Short Exposures to an Extremely Low-Frequency Magnetic Field (ELF MF) Enhance Protein but not mRNA Alkaline Phosphatase Expression in Human Osteosarcoma Cells

**DOI:** 10.2174/1874091X01812010065

**Published:** 2018-04-17

**Authors:** Tania Rescigno, Anna Capasso, Bruno Bisceglia, Mario Felice Tecce

**Affiliations:** Department of Pharmacy, University of Salerno, Fisciano (SA), Italy

## Abstract

**Background:**

Among electromagnetic fields treatments used in orthopedics, extremely low-frequency magnetic fields (ELF MF) need more detailed information about the molecular mechanisms of their effects and exposure conditions.

**Objective:**

Evaluation of the effects of an ELF MF exposure system, recently introduced among current clinical treatments for fracture healing and other bone diseases, on Alkaline Phosphatase (ALP) activity and expression in a human osteosarcoma cell line (SaOS-2), as marker typically associated to osteogenesis and bone tissue regeneration.

**Method:**

Cells were exposed to the ELF MF physical stimulus (75 Hz, 1.5 mT) for 1h. Cell viability, enzymatic activity, protein and mRNA expression of alkaline phosphatase were then measured at different times after exposure (0, 4 and 24 h).

**Results:**

Data demonstrate that this signal is active on an osteogenic process already one hour after exposure. Treatment was, in fact, capable, even after an exposure shorter than those commonly used in clinical applications, to significantly up-regulate alkaline phosphatase enzymatic activity. This regulation is produced essentially through an increase of ALP protein level, without changes of its mRNA concentration, while assessed magnetic field did not affect cell growth and viability and did not produce temperature variations.

**Conclusion:**

Tested low-frequency magnetic field affects cellular ALP expression with a posttranslational mechanism, without the involvement of regulations at gene transcription and mRNA level. This molecular effect is likely produced even within treated tissues during therapies with this signal and may be implicated in the induction of observed effects in treated patients.

## 1 INTRODUCTION

Practical use of Electro-magnetic Fields (EMF) in the treatment of several pathological conditions, including bone diseases, has attracted the attention of recent literature and clinical applications of electro-magnetic stimulation are becoming frequent [1-3]. Pulsed Electro-Magnetic Fields (PEMF) have been widely used for bone disorders treatments in animals and in humans [4-6]. There are also several pieces of evidence of PEMF potential applications in the treatment of osteoporosis [7, 8]. Extremely Low-Frequency (ELF) Electro-Magnetic Fields (EMF), along with pulsed EMF, have been shown to positively affect osteogenic processes [9-11]. Furthermore, it has been long known also the low intensity direct current ability to stimulate bone growth and remodeling [12-14].

Knowledge of molecular mechanisms underlying osteogenic effects of these signals appears very important for their appropriate clinical applications. PEMF seem to affect osteogenesis stimulating proliferation and differentiation of bone cells *in vitro*, increasing bone marker genes expression and inducing bone mineralization processes [15, 16]. ELF-MF showed to affect bone remodeling suppressing reabsorption and promoting the formation of bone tissue [3], while Capacitively Coupled Electric Fields (CCEF) have been proven to accelerate proliferation of osteoblast-like primary cells and to increase bone extracellular matrix formation *in vitro* [17, 18].

Alkaline phosphatase (ALP) activity and expression are typically associated markers of osteogenesis and bone tissue regeneration [19, 20]. This homodimeric enzyme, which is linked to cell membrane through Glycosylphosphatidylinositol (GPI), has four different forms: Tissue-nonspecific (TNAP), intestinal, placental, and germ cell [21, 22]. TNAP is ubiquitously expressed in many tissues, including liver, bone, and kidney, and is also referred as the liver/bone/kidney type [23, 21]. It contributes to bone tissue formation both increasing local concentration of inorganic phosphate (P_i_) and decreasing concentration of extracellular Pyrophosphate (PP_i_) [21, 20]. Within mineralization processes, TNAP hydrolyzes PP_i_, providing P_i_ for the hydroxyapatite formation.

Several studies demonstrated that cell ALP activity and expression may be affected by prolonged exposure to specific PEMFs in human mesenchymal stem cells [24, 11]. We previously reported the effects of a low-frequency (LF) Electric Field (EF), generated by an apparatus of current clinical use (frequency 60kHz, frequency of the modulating signal 12.5 Hz, 50% duty cycle, peak-to-peak voltage 24.5 V), on ALP activity [25]. Our signal induced a significant increase of alkaline phosphatase enzymatic activity, in two different cell lines (bone SaOS-2 and liver HepG2), already after one-h exposure [25].

In the current study, we analyzed the effects of one-h exposure, using an extremely low-frequency magnetic (75 Hz, 1.5 mT), on ALP enzymatic activity and expression in cultured human SaOS-2 cells. This exposure was also tested in the presence of three-dimensional collagen scaffolds. The magnetic stimulus was obtained by a recently introduced device for clinical treatments requiring bone regeneration.

## 2 MATERIALS AND METHODS

### 2.1 Cell Cultures and Treatments

Human osteosarcoma SaOS-2 cells [26, 27] and human breast cancer cell lines MCF-7 [28, 29] and SK-BR-3 [30, 31] were cultured in Dulbecco’s modified Eagle’s medium, with 4.5 g/L glucose with L-glutamine (Bio-Whittaker, Frederick, MD), 10% (v/v) fetal bovine serum (Sigma-Aldrich, St. Louis, MO), 100 mU/ml penicillin, and 100 mg/ml streptomicyn (Bio-Whittaker) at 37ºC with an atmosphere of 5% CO_2_.

Cells were seeded at a density of 1x10^5^ in each well of 24-well tissue culture plates and allowed to adhere for 24 h. Cells were then exposed for 1 h to the magnetic field (75 Hz, 1.5 mT) at room temperature (22ºC). Simultaneously with the exposure, under the same experimental conditions, negative control received no treatments: identical culture plates containing identical cell numbers were kept in the same laboratory for the same 1 h time in an identical installation without magnetic exposure. Cell viability, ALP activity, mRNA and protein level were assayed at 0 h, 4 h, and 24 h after exposure.

### 2.2 ELF MF Exposure system

Generator system and field strength measures were fully described in previous studies [32, 33]. It consists of a pair of coils of copper wire, placed opposite to each other and in a signal generator. Multiwell plates were placed in a Plexiglass holder between this pair of coils so that the plane of the coils was perpendicular to multiwell plates. Generator system (75 Hz, 1.5 mT) and holder were kindly provided from IGEA (Carpi, Italy). This winding results in a uniform magnetic field between coils with the primary component parallel to the axis of the two coils. This uniform field results from the sum of the two field components parallel to the axis of the coils and the difference between the components perpendicular to the same axis. This installation allowed to perform controlled exposures of cells attached to the bottom of each well of a 24-well tissue culture plate (well diameter, 15.6 mm; well volume, 3.4 ml). Fig. (**1**), panel A shows the schematic geometry of multiwells in a simulation model (Comsol MultiphysicsTM) (growth area 1.9 cm^2^). Fig. (**1**), panel B shows a computer modeling of the magnetic field between the coils (Comsol MultiphysicsTM). Arrows indicate magnetic field (H) strength and direction, demonstrating a constant distribution within the whole multiwell plate. Fig. (**1**), panel C shows a picture of the exposure system with the Plexiglass holder keeping multiwell plate between coils.

### 2.3 Measurement of Temperature of Culture Medium

Culture medium was added to each well of two 24-well tissue culture. As for the exposure experiment, one plate was exposed to the ELF MF system and the other one (control) received no treatment. The temperature of exposed and unexposed medium was then measured several times (at 0, 15, 30, 45, and 60 min), using an electronic contact thermometer (Sigma-Aldrich, St. Louis, MO). Each measurement was carried out on at least eight different detections and four different experimental sessions.

### 2.4 3D Scaffolds

Collagen scaffolds employed for our investigation were kindly provided by Finceramica (Faenza, Italy). Scaffolds were produced by a process integrating an organic compound (collagen type I) with bio-active hydroxyapatite nano-crystals with magnesium salts. For treatments, cells were layered onto cylindrical scaffolds (5 mm height, 6 mm diameter) covered with standard medium, within 24-well tissue culture plates. Exposure treatments were then performed after 24h. Cells were exposed for 1 h in the same conditions described for other treatments.

### 2.5 Scanning Electron Microscopy (SEM)

SaOS-2 cells were seeded at a density of 1x10^6^ in each well of 24-well tissue culture plates in presence of one cylindrical collagen scaffold and allowed to adhere for 24 h. Later, scaffolds colonized by cells were fixed with glutaraldehyde, dehydrated with alcohol and dried with a critical point dryer (mod. K850; Quorum Technologies Ltd., East Sussex, United Kingdom). Each sample was then metalized with gold and observed by a scanning electron microscope equipped with a transmission electron microscopy detector (S-TEM, mod. Zeiss Ultra Plus; Carl Zeiss SMT AG, Oberkochen, Germany).

### 2.6 MTT and ALP Activity Assays

The Methyl Thiazolyl blue Tetrazolium (MTT) spectrophotometric dye assay was used to detect cell proliferation ability, which is proportional to cell number, being an assay of mitochondrial metabolic activity. After removal of medium, cells were incubated for 2 h in 1 mL of 1mg/mL MTT (Sigma-Aldrich) at 37°C. Formazan crystals were then solubilized adding into each well 800 μL of dimethyl sulfoxide and absorbance was detected at 490 nm with a microplate reader (Thermo Scientific, Waltham, MA).

ALP activity assay was performed by a colorimetric reaction [25]. Cells were incubated in a lysis solution containing 0.1% Triton-X 100 (Sigma-Aldrich), 50 mM citric acid (Sigma-Aldrich) (pH 5.5), and 5 mM p-nitrophenyl phosphate (p-NPP) (Sigma-Aldrich), for 45 min at 37°C. During this time, ALP enzyme catalyses the hydrolysis of p-NPP into p-nitrophenol (p-NP). The reaction was stopped with 1 M NaOH and the p-NP production, proportional to the amount of enzyme activity, evaluated by measuring absorbance at 405 nm. The results were normalized for cell number.

Each test was carried out on at least eight cell extract samples for each experimental point from three full experimental replicates.

### 2.7 Cell Protein Extraction and Western Blot Analyses

After treatments, cells were washed and lysed as previously described [34]. Twenty micrograms of total protein extract were separated by 12% SDS-polyacrylamide gels and transferred onto nitrocellulose membranes (GE Healthcare Milano, Italy) in a cooling system at 100 V for 1 h. Membranes were saturated for 1 h at room temperature with 0.1% Tween-20 (Sigma-Aldrich), 5% dry milk (Bio-Rad Laboratories, Hercules, CA) in PBS. Membranes were then incubated with an antibody against human tissue non-specific alkaline phosphatase (Abcam, Cambridge, UK) diluted 1:10,000, overnight at 4°C, washed several times and incubated with peroxidase-conjugated secondary anti-rabbit antibody (Santa Cruz Biotechnology, Santa Cruz, CA), diluted 1:10,000, for 1 h at room temperature. Specific bands were then detected by ECL Western blot system (GE Healthcare). Antibody against GAPDH (Santa Cruz Biotechnology) were used to perform a normalizing control. Densitometry of bands was performed with ImageJ software [http://rsbweb.nih.gov/ij/download.html]. Molecular sizes were evaluated referring to protein molecular weight standards (Bio-Rad Laboratories). Each treatment and analysis was performed at least in triplicate separate experiments.

### 2.8 RNA Extraction and Reverse-Transcription

Total RNA was isolated from exposed and not exposed SaOS-2 cells as previously described [34].

### 2.9 Real-Time PCR

Real-time PCR was performed with a Light-Cycler 480 (Roche Diagnostics, Mannheim, Germany) using SYBR Green detection in a total volume of 20 μl with 1 μl of forward and reverse primers (10 mM) (Primm, Milan, Italy) and 10 μl of SYBR Green I Master-Mix (Roche Applied Science, Mannheim, Germany). Reactions included an initial cycle at 95°C for 10 min, followed by 40 cycles of denaturation at 95°C for 10 sec, annealing at 56°C for 5 sec, extension at 72°C for 15 sec.

To analyze ALP expression level, the following primer sets were used: forward ALP 5’-GAA CGT ATT TCT CCA GAC CC -3’; reverse ALP 5’-AAA GAC CTC AAC TCC CCT GA -3’ (designed on human liver/bone/kidney-type alkaline phosphatase (ALPL) gene sequence, GenBank: AH005272.1); forward 18S 5’-CGA TGCTCT TAG CTG AGT GT -3’; reverse 18S 5’-GGT CCA AGA ATT TCA CCT CT -3’.

Fold change of induction was determined by calculating the ratio between control and treatment normalized signals. Each treatment was performed at least in triplicate separate experiments.

### 2.10 Statistical Analysis

Data are presented as mean ± standard deviations. Differences between exposed and unexposed cells were analyzed by Student t-test. Differences were considered significant with p<0.05.

## 3 RESULTS

### 3.1 Cell Viability After ELF MF Exposure

**Fig. (2):**
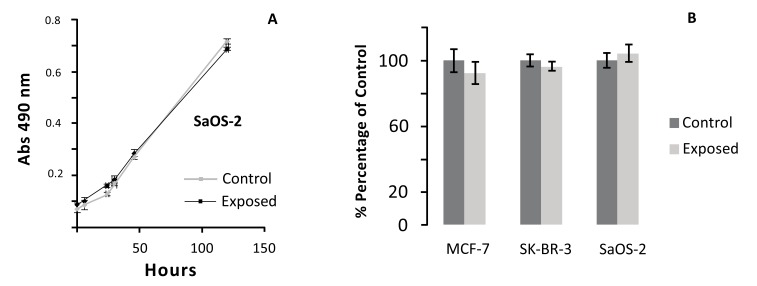
Viability of cultured cells after 1 h exposure with ELF MF as determined with MTT assay. (A) Viability time course of control and exposed SaOS-2 cells. (B) MCF-7, SK-BR-3, and SaOS-2 viable cells immediately after MF stimulation, reported as percentage of viability of unexposed control cells. Reported values are means of three independent experiments ± standard deviations.

We first examined ELF MF effects on cell viability by MTT assay. As MTT assay is proportional to cell metabolism, values are proportional to proliferation and viability. SaOS-2 cells were treated with ELF MF for 1 h and MTT assay was performed at different times (0, 6, 24, 30, 46, 52, and 144 h) after exposure. As shown in Fig. (**2A**), there are no significant differences between control and exposed cells at each assayed time. Meanwhile, total cell number tends to increase with the time as cell growth proceeds. Moreover, our treatment did not affect also the viability of two breast cancer cell lines (MCF-7 and SK-BR-3) when assayed immediately after 1 h of ELF MF exposure (Fig. **2B**). While our work was mostly aimed to study the effect of the apparatus on cell related to bone tissues, the use of MCF-7 and SK-BR-3, both derived from breast cancers, but with different degree of malignancy was intended to test our signal also with different cell lines, essentially also showing also in this case absence of toxicity. Preliminary observations indicate similar absence of effects also on Hep G2 cells (not shown).

### 3.2 Effect of ELF MF on Culture Medium Temperature

In order to check for possible thermal effects of our signal, temperature of culture medium was measured several times (0, 15, 30, 45, and 60 min) during exposure and compared with that of unexposed culture medium. Detected temperatures of control and exposed samples are plotted in Fig. (**3**). It can be seen that there are essentially no temperature differences between unexposed and exposed culture medium at each analyzed time (until 60 min). Thus, exposure did not produce any heating phenomenon which could be involved in observed results.

### 3.3 Alkaline Phosphatase (ALP) Enzymatic Activity After ELF MF Exposure

To evaluate ELF MF exposure ability to increase osteogenesis, alkaline phosphatase enzymatic activity was measured as a marker of the early stages of this process. Fig. (**4**) shows the results of p-NPP assays performed on SaOS-2 cells immediately, 4, and 24 h after one h exposures. Magnetic stimulation was able to induce a significant increase of ALP activity in exposed cells compared to control cells. In particular, we observed the highest differential value 4 h after exposure (about 35% increase), while both immediately and 24 h after treatment smaller differences (about 20% increase) were observed. Since exposure does not affect cell numbers even after a very large number of hours, as shown in Fig. (**2**), it was not necessary to correct for cell numbers.

### 3.4 Alkaline Phosphatase (ALP) Protein and mRNA Expression After ELF MF Exposure

To verify if increases obtained with p-NPP assays after ELF MF exposure were due to an increase of ALP intrinsic enzymatic activity or of its protein expression levels, Western blot analyses were carried out. Protein levels of tissue non-specific alkaline phosphatase were analyzed in control and treated SaOS-2 cells at the same previous assayed times after exposure. This ALP isozyme form is the one specifically present in bone and also in numerous other tissues. Fig. (**5**) shows as stimulated cells express an higher level of ALP protein immediately after treatment and even more at 4 and 24 h. All bands in the upper panel of Fig. (**5**) stand for ALP, as this protein migrates variably according to its phosphorylation, glycosylation and lipid level [21]. In particular, densitometric analysis of bands reveals about 40% increase of ALP expression at 0 h after exposure, which becomes about 150% at longer times.

Real-time PCR analyses were performed to evaluate ELF MF exposure ability to affect alkaline phosphatase expression also at mRNA level. Total RNA was extracted from SaOS-2 control and exposed cells and ALP mRNA level was determined 0, 4, and 24 h after exposure. As shown in Fig. (**6**), the magnetic field produced by our apparatus does not significantly affect ALP expression at mRNA level in all cases.

### 3.5 ALP Activity after ELF MF Exposure in Cells Seeded with Collagen Scaffolds

ALP enzymatic activity was assayed also in cultured SaOS-2 cells layered onto the surface and within the 3D structure of collagen scaffolds, imitating bone tissue organization, and exposed for 1 h to ELF MF. Fig. (**7**) reports results of p-NPP assays performed on cells colonizing collagen scaffolds 0, 4 and 24 h after MF exposure. Exposition does not seem to affect cell numbers even after a very large number of hours and no corrections for cell numbers were performed. Nevertheless, reproducible MTT assay was not technically feasible on cells cultured within scaffolds and we had to assume that cell viability within scaffolds was similarly unaffected by exposition as in common culture. Fig. (**8**) shows that exposed cells layered onto scaffolds were able to colonize them inside, as assessed by scanning electron microscope analysis. Similar images were also obtained with unexposed cells (not shown). Again, stimulation significantly increased alkaline phosphatase activity and this increase appears even larger than those obtained in absence of collagen scaffolds. Control cells show decreased activity at 4 and 24 h and this could derive from specific effects of scaffold culture conditions.

## 4 DISCUSSION

Different physical stimuli, such as PEMF, ELF EMF, and CCEF, were reported to positively affect osteogenic processes [12, 10, 14, 11]. These observations promoted the development of several devices to be used in clinical practice for bone diseases therapeutic treatment [25, 35, 10]. Several studies identified also some molecular mechanisms underlying the beneficial effects of these signals on bone mineralization and remodeling. This is very important in the perspective to employ more properly electro-magnetic fields for treatment of fractures and other bone damages. PEMF exposure increased mRNA amount of many factors involved in bone growth, protection and remodeling and enhanced deposition of calcium and other extracellular matrix components in human bone marrow mesenchymal stem cells [36]. ELF-EMF was demonstrated to prevent spinal cord injury-induced osteoporosis, suppressing bone reabsorption and inducing new tissue formation [3]. Hartig *et al*. [17] found that CCEF exposure of osteoblast-like primary cells enhanced cell proliferation and synthesis and secretion of extracellular matrix-related proteins.

In most cases, the ability of these magnetic or electric stimulations was found to increase the enzymatic activity and/or protein expression of alkaline phosphatase [24, 11], which was selected as a marker of bone regeneration processes [19, 20]. In our previous work, we found significant increases of ALP activity, in two different human cell lines, after one-h exposure to a low-frequency electric field from an apparatus used in clinical therapy [25] and this signal did not significantly affect gene expression, suggesting that clinical applications of this signal should not produce cytotossicity, genotoxicity and mutagenesis [37].

In the present study, we show the effects of short exposures to an Extremely Low Frequency Magnetic Field (ELF MF) on human osteosarcoma cell line SaOS-2, which is an effective and widely used *in vitro* model of human bone tissue [26]. SaOS-2 is a kind of osteoblast-like cell and widely used in the study of osteogenesis, expecially to analyze the effect of electromagnetic signals [38, 39]. The wave was generated by a device recently introduced among clinical treatments of fractures or other bone diseases. One-h exposure condition was selected as similar to those used for clinical treatments with the apparatus. This treatment did not affect cell proliferation and viability of bone derived SaOS-2 and also of two breast cell lines, indicating a similar effect also on cells from a different tissue. Since clinical treatments are aimed on tissues including many different cell types, this allows to exclude, at least at preliminary level, relevant toxic effects on cell health and proliferation.

There is a wide literature on the applications of electromagnetic fields in the treatment of bone diseases and osteogenesis, both indicating interesting perspectives and need of further investigations [1-12, 40-43]. Several investigations have already analyzed the molecular effects of longer exposure times to some signals comparable to that used in this work [44-49]. In view of the difficulties, in clinical setting, related to so prolonged exposures (from 1 to 22 days), we considered relevant to obtain also data on biological effects and molecular mechanisms of a shorter term exposure (1 h) to the ELF MF signal. In addition, to verify if responses to our stimulation were early or late, and if they were lasting over time, each ALP analysis was performed immediately, 4, and 24 h after exposure. Differently as noted in other studies [50], viability and proliferation of SaOS-2 cells, and of two other different cell lines (MCF-7, SK-BR-3) were not affected from the 1h-exposure to our system. This excludes the possibility that observed ALP variations were related to cell amounts and other general conditions. Using room temperature to perform exposures is certainly an approximation, but this is the best one to assure the highest reproducibility to avoid interference from the temperature and CO_2_ controls of eukaryotic cell incubators, while identified mechanisms in these conditions should also be likely active within complete living organism, although, as usually with data using experimental models with cultured cells, this may have to be fully confirmed through further research. Observed effects on ALP cannot be consequent to a thermal effect of ELF MF exposure since no temperature variations were essentially detected.

We found significant increases of ALP activity and expression in 1 h ELF MF-exposed SaOS-2 cells. Alkaline phosphatase activity and protein expression increased immediately after treatment and even more at 4 h. In particular, we analyzed mRNA and protein expression of bone ALP isozyme, the tissue-nonspecific ALP (TNAP). Increases of protein expression were higher than those observed for enzymatic activity of ALP. Increases of ALP enzymatic activity (about 20% fold increase) and protein expression (about 40% fold increase) resulted comparable immediately after 1 h exposure. However, at 4 and 24 h increase rates become very different; at these times enzymatic activity increases were about 35% and 20% respectively, while those of ALP protein were much higher (about 150% increase). Since essentially no ALP mRNA variations were observed, this means that the increases of ALP enzymatic activity following treatment with ELF EMF are just consequent to the increase of the ALP protein concentration. Furthermore, the fact that the increases in ALP activity and expression were still evident after 24 h suggests that ELF MF effects on the enzyme activity and expression last over time. The higher level of enzymatic activity in control samples at 24 h can be attributed to the normal proliferation increase of cells number, being essentially proportional to it, while increases due to ELF MF exposure produce a significantly higher ALP value at each time point.

Magnetic field exposure increased ALP protein expression without changing its mRNA level at all tested times. Therefore, our treatment, rather than acting at transcriptional level, likely affects post-transcriptional, post-translational or degradation regulation mechanisms of ALP expression, affecting either mRNA translation efficiency and/or half-life protein stability. While our data do not allow to indicate which mechanism may be responsible for this regulation, there are several pathways described in the literature which could provide this effect [51].The fact that the effect does not affect mRNA expression allows also to exclude the possibility of genotoxic effects.

Moreover, we demonstrated that a short-term exposure to our signal caused a significant and lasting increase of both ALP enzymatic activity and protein expression, without affecting cell viability and proliferation. In fact, in the same experimental conditions, MTT analysis showed no significant differences in SaOS-2 cell number between control and exposed cells, even 144 h after 1 h-exposure. These data confirm that ELF MF treatments, within analyzed conditions which are also similar to those used in clinical treatments, do not cause cytotoxicity, do not interfere with cell growth and do not produce direct effects on gene regulation.

**Fig. (8):**
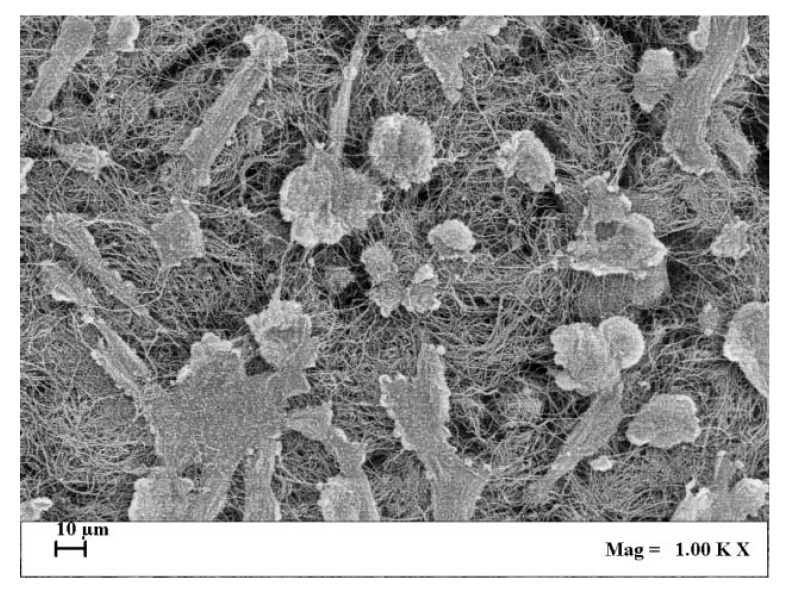
SEM images of SaOS-2 cells cultured inside collagen scaffold for 24 h, after exposure to ELF MF.

The signal of our apparatus was also tested on SaOS-2 cells within 3D scaffolds, which are aimed to physically improve replacement of damaged tissues within surgical processes [52]. These scaffolds were structured trying to mimic bone tissue morphological organization. Cells layered onto scaffolds were able to colonize them inside as verified through scanning electron microscope analysis (Fig. **8**). In experimental conditions similar to previous analyses, we also found a significant increase of alkaline phosphatase activity, mainly 4 and 24 h after exposure, again with no effect on cell viability. Control ALP activity results lower in cells cultured within scaffolds, especially after 4 h and this might be consequent to the different cell culture conditions. Comparing these results with those obtained without scaffolds, the stimulatory effect on the ALP activity induced by examined magnetic field is stronger. This may mean that within scaffolds, with the different microenvironment around cultured cells, exposure can induce improved changes of the activity and/or protein expression of this membrane enzyme and possibly better regeneration effects on tissues.

## CONCLUSION

These data demonstrate relevant ELF MF effects on cellular ALP expression and, considering the role of this enzyme in these processes, provide additional information to validate the exposure to these signals as a procedure to increase osteogenesis in clinical treatments [19, 20]. This is also supported by the fact that these effects resulted evident and significant already 1 h after exposure, which is a time period similar to those used in these clinical applications. Moreover, we firstly highlighted an ELF MF-induced modulation of ALP protein expression, with a posttranslational mechanism without the involvement of regulations at transcriptional and translational level, which are not directly affected from the used physical signal.

## Figures and Tables

**Fig. (1):**
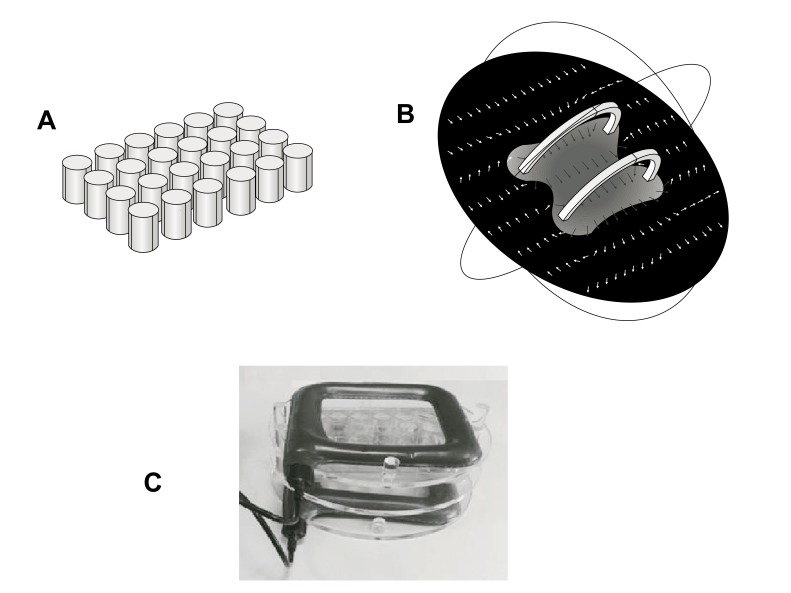
Description of ELF MF exposure system. Panel A shows the schematic geometry of multiwells used in a simulation model. Panel B shows a computer modeling of the magnetic field between coils, with arrows indicating magnetic field (H) strength and direction, demonstrating a constant distribution within whole multiwell plate. Panel C shows a picture of the exposure system with the Plexiglass holder keeping multiwell plate between coils.

**Fig. (3):**
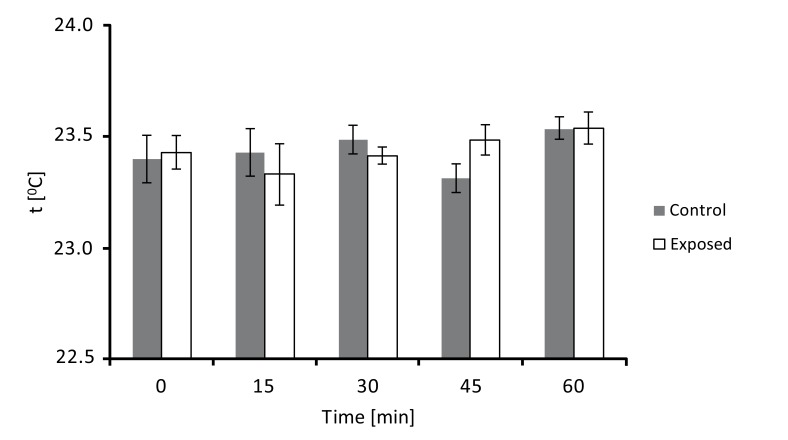
Temperature monitoring of culture medium during exposure. Culture medium was exposed or not to ELF MF and temperature (°C) measured every 15 min. Reported values are means of three independent experiments ± standard deviations.

**Fig. (4):**
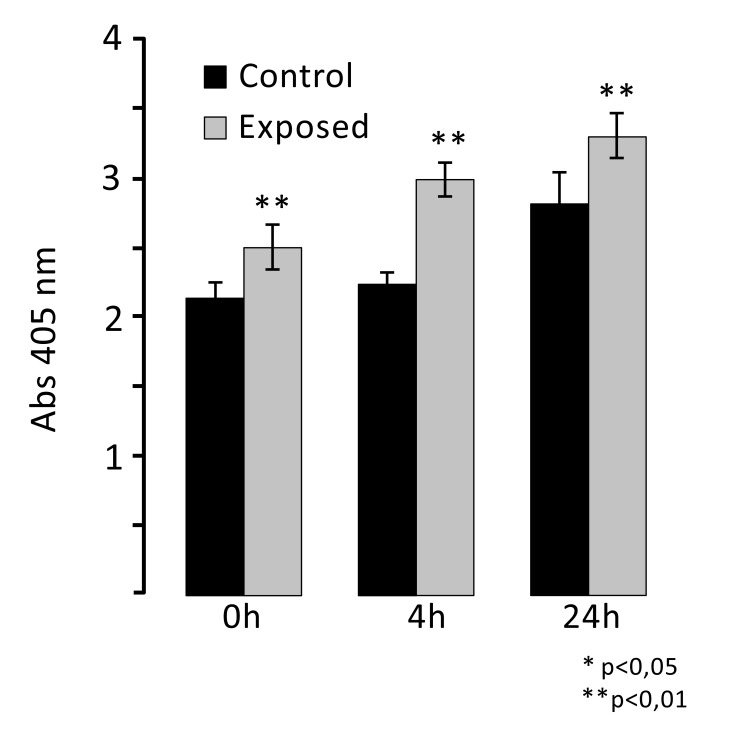
ELF MF exposure increases alkaline phosphatase activity. SaOS-2 cells were exposed or not to ELF MF for 1 h and after 0, 4, and 24 h, ALP activity assays were carried out. Values are reported as 405 nm absorbance and are expressed as means of three independent experiments (each carried out with at least eight assay replicates) ± standard deviations.

**Fig. (5):**
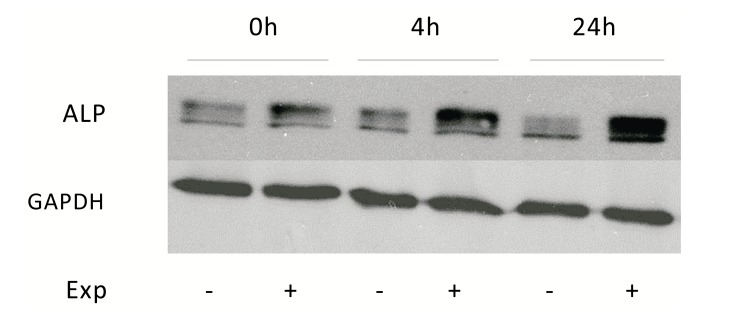
ELF MF exposure increases alkaline phosphatase protein levels. SaOS-2 cells were exposed or not to ELF MF for 1 h and after 0, 4, and 24 h, were lysed. ALP protein levels were determined by Western Blot analysis. Densitometric analysis of bands reveals about 40% increase of ALP expression at 0 h after exposure, which becomes about 150% at longer times. Glyceraldehyde 3-Phosphate Dehydrogenase (GAPDH) was used as normalization reference. Images shown are representative of three independent experiments.

**Fig. (6):**
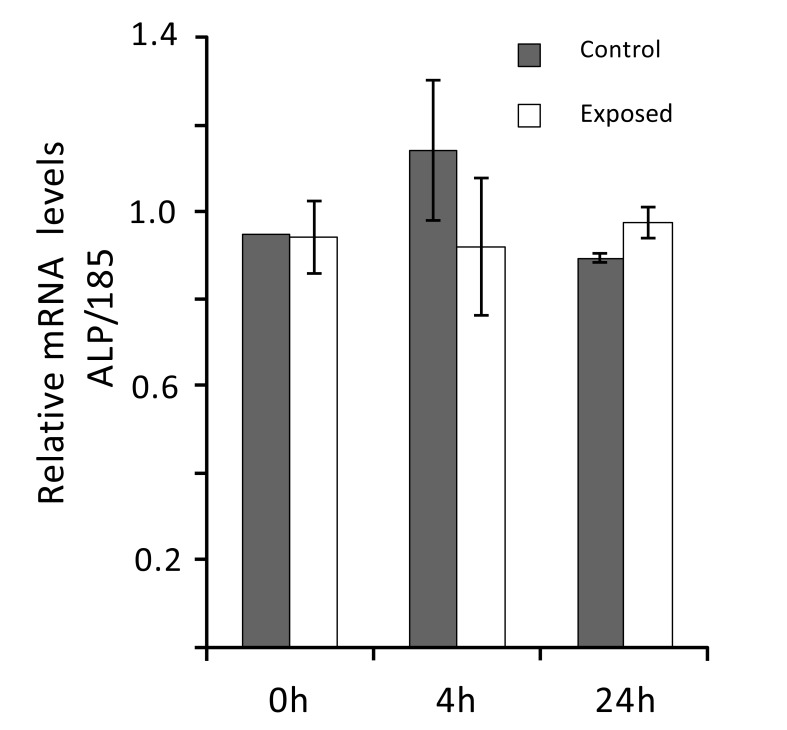
Alkaline phosphatase mRNA levels are not affected by ELF MF exposure. SaOS-2 cells were exposed or not to ELF MF for 1 h and after 0, 4, and 24 h, total mRNA was extracted. ALP mRNA levels were determined by Real-time PCR calculating ratios between 18S normalized signals from exposed and control cells. Reported data were expressed as means of three independent experiments ± standard deviations.

**Fig. (7):**
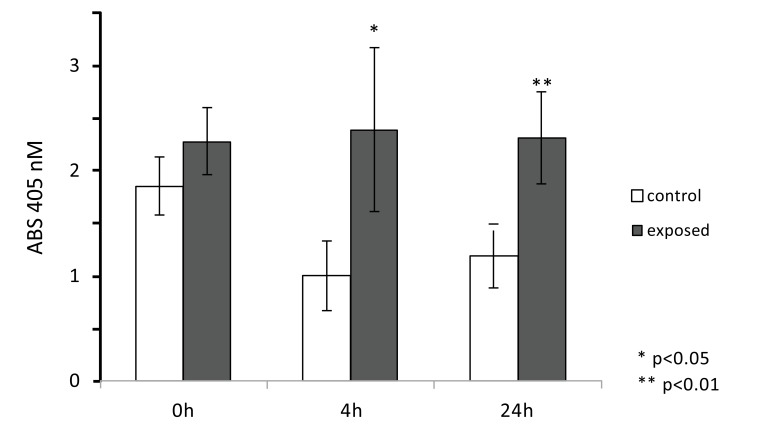
Alkaline phosphatase activity of SaOS-2 cells colonizing collagen scaffolds at 0, 4 and 24 h, after 1 h ELF MF exposure. Values were measured as 405 nm absorbance and were expressed as means of three independent experiments (each carried out with at least eight cell extract sample replicates) ± standard deviations.
